# Biobanking from the patient perspective

**DOI:** 10.1186/s40900-015-0001-z

**Published:** 2015-06-25

**Authors:** Derick Mitchell, Jan Geissler, Alison Parry-Jones, Hans Keulen, Doris C. Schmitt, Rosaria Vavassori, Balwir Matharoo-Ball

**Affiliations:** 1 EU Joint Programme—Neurodegenerative Disease Research (JPND), 1st floor, Liffey Trust Building, 117-126 Upper Sheriff St., Dublin, Ireland; 2 Leukemia Patient Advocates Foundation, Bern, Switzerland; 3 European Patients’ Academy on Therapeutic Innovation (EUPATI), Am Rothenanger 1b, 85521 Riemerling, Germany; 4 Wales Cancer Bank, Cardiff University, Room 273, B-C link corridor, 2nd Floor, UHW Main Building, Heath Park, Cardiff, CF14 4XN UK; 5 Chordoma Foundation, PO Box 2127, Durham, NC 27702 USA; 6 Foundation PATH, Munich, Germany; 7 Italian Association for Alternating Hemiplegia of Childhood, Verderio (LC), Italy; 8 Biobank and Clinical Registry for Alternating Hemiplegia of Childhood, Verderio (LC), Italy; 9 International Consortium for the Research on Alternating Hemiplegia of Childhood, Lyon, France; 10 Nottingham Health Science Biobank, EMPATH Pathology Services, Nottingham University Hospital NHS Trust, City Campus, Nottingham, UK; 11 The David Evans Medical Research Centre, Hucknall Road, Nottingham, NG5 1PB UK

**Keywords:** Biobank, Partnering, Research, Patient, Involvement

## Abstract

**Plain English summary:**

Biobanks are collections of donations of biological material (DNA, cells, tissue etc.) and related data which are very valuable for research into human diseases. A variety of biobanks exist for example within hospitals, research institutes, pharmaceutical companies and patient organisations. The role of patients in biobanking is changing from being seen simply as donors, to actual collaborators in the design, development and the running of biobanks. In this article, we provide a number of examples of patients acting as partners at the heart of biobanking, where their voice and perspective is being seen and used as a valuable resource for the biobank. Our aim is that these examples can be used by those who work with patients in biobank-based research, to design future strategies for patient and public involvement in all biobanks.

**Abstract:**

Biobanks and biobanking research plays an increasingly important role in healthcare research and delivery as health systems become more patient-centred and medicine becomes more personalised. There is also growing acceptance and appreciation of the value that patients, patient advocacy organisations and the public can bring as stakeholders in biobanking and more generally in research. Therefore, the importance of active, early and sustained engagement and involvement of patient and public representatives in biobanks will become increasingly relevant.

Organising and facilitating patient and public involvement in biobanking takes considerable time and effort for all stakeholders involved. Therefore, for any biobank operator considering involving patients and the public in their biobanking activities, consideration of best practices, current guidance, ethical issues and evaluation of involvement will be important.

In this article, we demonstrate that patients are much more than donors to biobanks—they are collaborators at the heart of biobanking with an important voice to identify perspective, which can be an extremely valuable resource for all biobanks to utilise. The case studies herein provide examples of good practice of patient involvement in biobanking as well as outcomes from these practices, and lessons learned. Our aim is to provide useful insights from these efforts and potential future strategies for the multiple stakeholders that work with patients and the public involved in biobank-based research.

## Introduction

Biobanks collect, process, store and distribute biological material and related data to research organisations. These “biospecimens” and data are used by scientists to learn more about human diseases, their causes and their effects and to develop better prevention measures, better diagnostic tests and better therapies [1]. Different types of biobanks exist and are described in greater detail in the “Definitions” section below. Unless otherwise stated, the term “biobank” in this article refers to disease-oriented, general biobanks comprising of human tissue, cell or DNA biosamples and associated clinical data (see “Definitions” section).

Biobanks do not operate in isolation. They exist within a diverse “ecosystem” of stakeholders which includes the public, patients, healthcare workers, scientists, government, funders, healthcare providers, ethicists, regulators and others. The sheer variety of stakeholders involved in maintaining biobanks reflects the diversity of biobanks themselves [2]. Biobanking operators such as hospitals, research institutes, pharmaceutical companies and patient organisations interpret their own biobanking activities differently, very often according to the background of the founding organisation and the particular context in which the biobank is embedded (e.g. geographical location, ability to network nationally or internationally etc.).

The role of patients and the public in biobanking activities has been viewed traditionally as biobank participants rather than as collaborators in the design, development and ongoing operation and governance of biobanks. However, there is growing acceptance and appreciation of the value that patients, patient advocacy organisations and the public can bring as stakeholders in biobanking and more generally in research [3, 4].

This article demonstrates the wealth of experience that now exists within patient communities which makes them valuable partners and sources of input in biobanking research. We demonstrate that patients are much more than donors—they are collaborators at the heart of biobanking with an important voice and a unique perspective, which can be an extremely valuable resource for all biobanks to utilise. The case studies herein provide examples of good practice of patient involvement in biobanking as well as outcomes from these practices, and lessons learned. Our aim is to provide useful insights from these efforts and potential future strategies for the multiple stakeholders that work with patients and the public involved in biobank-based research.

## Definitions

The term “patient” in this article refers to the patient, his/her relatives or patient advocates as collaborative or representative voice of patients.

The term “patient involvement” in this article refers to “involvement in research” (i.e. being actively involved in the research process itself, rather than being participants or subjects of the research).

The term “patient organisation” in this article refers to not-for-profit organisations which are patient focused, and whereby patients and/or carers (the latter when patients are unable to represent themselves) represent a majority of members in governing bodies. These could be either general umbrella organisations (e.g. representing either specific disease organisations and/or national umbrella organisations) or disease-specific organisations (i.e. representing national organisations or individual patients on acute and/or chronic diseases).

The term “biobank” generally refers to a service whereby biological materials and the data associated with those materials are collected, stored, processed and distributed for the purposes of scientific research and/or medical treatment. Typically, those “biological materials” are human samples—such as tissue, blood, body fluids etc., and the “data” is any information, including medical information pertaining to the donor of that sample. Biobanks exist within a variety of institutions, including academic medical institutions, and pharmaceutical and biotechnology companies. They can also be stand-alone organisations, including independent companies (both for profit and non-profit) that can provide biobanking services and access to samples as a service to the research community or patients. There are multiple designs according to different possible goals. Briefly, human sample-related biobanks include three major types:

- Population biobanks: whose primary goal is to obtain biomarkers of population identity and susceptibility, typically from the DNA of large numbers of healthy donors, representative of a country/region/ethnic cohort.
- Disease-oriented biobanks for epidemiology: whose activity is focused on biomarkers of exposure, using large numbers of samples, usually following a healthy exposed cohort/case-control design, and studying germ-line DNA or serum markers and large amounts of specifically designed and collected data.
- Disease-oriented general biobanks (e.g. tumour banks): where their goals correspond to biomarkers of disease through collections of biosamples and their derivates (DNA/RNA/proteins), usually associated to clinical data and sometimes associated to clinical trials. The amount of clinical data linked to the sample determinate the availability and biological value of the sample.

Unless otherwise stated, the term “biobank” in this article refers to disease-oriented general biobanks, comprising of human tissue, cell or DNA biosamples and associated clinical data.

## Main theories and background

### Towards a patient-centred approach to healthcare—role of biobanking

Directly involving patients and the public in shaping both the organisation and delivery of healthcare services is central to current health reform agendas around the world. Realigning public services based on evidence from service user’s experiences, and designed with and by the public rather than on their behalf, is challenging national healthcare systems, with particular examples emerging in the USA and UK [5, 6]. It is becoming clear that different types of involvement exist and approaches to involvement are different in those countries that adopt either a “rights-based” or a “regulatory” approach to healthcare [7].

Developing in parallel to patient-centred healthcare is the concept of personalised medicine [8]. In order to serve the needs of personalised medicine, biobanks and biobanking research will play an increasingly important role in healthcare research and delivery. It is predicted that the integration of biobanking at all levels of disease-specific, epidemiological and applied biomedical research and healthcare will increase progress into health research and decrease costs of both clinical trials and daily healthcare [9]. Treating patients and patient organisations as full partners in this process is key in moving towards a patient-centred care model, thus building the foundation for patients to take responsibility for managing their care and overall health [10].

### Evolution of patient involvement in research

Most health research would be impossible without the active involvement of patients. Traditionally, patients have been involved “passively” as subjects/participants in research, with patients and their representative organisations only rarely seen as partners in research practices. However, the last 20 years has seen a growing interest and activity at a local, national and international level in patient engagement and in particular a focused interest in patient and public involvement (PPI) in research, with projects in the European Union [11, 12], Australia [13], Canada [14], UK [15] and other countries, focusing on different aspects of PPI.

Many people describe public involvement in research as research that is done with or by the public, and not to, about, or for them [16]. Patients and the public have been “involved” or “engaged” in research in a variety of different ways. For example, this includes identifying and prioritising research topics, being part of research advisory groups and steering groups, undertaking research projects and reporting and communicating research findings. An obstacle to the involvement of patients is often the limited knowledge that patients have of the technical fields of research such as biobanking and in part to the limited understanding by researchers of how patients could contribute to research (and to biobanks). The idea of patient involvement can be difficult for researchers as it does not adhere to the traditional “scientific method”. The experiential knowledge of patients and the public, according to some, lacks the objectivity, verifiability, universality and rationality of scientific knowledge [17]. However, it is now increasingly appreciated among researchers that patients’ knowledge and experience is valuable for research and contributes to increasing the quality, relevance and appropriateness of research processes [3, 18]. PPI can also contribute to the broader democratisation of research, through participatory forms of involvement that encourage partnership in research [19–21]. This increased appreciation of early and sustained engagement and involvement of patients in research is captured by the following quote from Kish [22]:

Actually, it’s surprising that it has taken us this long to focus on patient engagement because the results we have thus far are nothing short of astounding. If patient engagement were a drug, it would be the blockbuster drug of the century and malpractice not to use it.

Leonard Kish—Principal and Co-Founder of VivaPhi

## Key messages

### Patient involvement in biobanking activities

For most patients and patient organisations, the primary objective for their involvement in biobanking activities is to find the cause of their disease or to improve their diagnosis and treatment options [23]. The degree of involvement can range from promoting the existence of a biobank to governing an entire biobank. When looked at over the last 20 years, this range of involvement has evolved through a number of important, overlapping phases:

#### I: Emergence of patient-led biobanks

Although an ever-increasing number of patient organisations are establishing their own biobank, the emergence of patient-led and patient-run biobanks began only relatively recently. Individual patients, and representative patient organisations, many unsatisfied with the speed of research into their respective conditions, established their own biobank, primarily to enable them to provide a greater contribution and influence on research in their disease area. Three case studies of patient-led biobanks in the USA (Chordoma Foundation Biobank), Germany (Patients’ Tumor Bank of Hope (PATH) Biobank) and Italy (Italian Biobank AHC) are provided in this article as examples in this regard. Another example worth highlighting is the Généthon DNA and Cell Bank driven by the French patient organisation Association Française contre les Myopathies (AFM). AFM become active in genetic research in the 1980s to help find a cure for neuromuscular diseases and founded the biobank in the mid-1990s [24]. The organisation has retained the decision-making power over the biobank, whilst maintaining a tight partnership with the scientists involved and is now one of the largest banks for genetic research in Europe [25].

Patient-led initiatives were also among the first to develop networked biobanking activities. The European network of DNA, cell and tissue biobanks for rare diseases (EuroBiobank) was the first pan-European network of biobanks—established in 2001 by the EURORDIS [26] and AFM patient organisations—and currently operated by the TREAT-NMD network. This network of 16 biobanks from eight countries successfully consulted with patient organisations in the construction of its ethical guidelines and consent forms. EuroBiobank continues to provide human DNA, cell and tissue samples as a service to the scientific community conducting research on rare diseases [27].

A further example is the patient-led “Genetic Alliance Biobank”, established in the USA in 2003 based on the organisational strategies of PXE International, an advocacy group set up by two parents whose children were diagnosed with the incurable condition PXE. The Genetic Alliance uses a cooperative, cost-sharing model that translates into a tightly controlled infrastructure for patient organisations to conduct biobanking activities. Genetic Alliance sets high standards for patient involvement in research, exceeding the requirements of applicable US federal, state and local laws, rules and regulations [28].

#### II: Establishing the patient position on biobanks

A number of European-wide initiatives have been able to develop “representative” patient positions on biobanking. The first clear recommendation was in 2006 by the European platform for patients’ organisations, science and industry (EPPOSI)—an EU patient-driven partnership between patient organisations, industry and academic science and clinicians. In the first conference to bring these different stakeholders together to discuss the future of biobanks, representatives of several patient organisations demonstrated i) how their self-developed biobanks contribute to progress to effective therapies for their diseases, ii) what key role patient groups play in promoting the need for biobanks and iii) how they contribute to raise awareness about the usefulness of biobanks and to accelerate the collection of precious biomaterial [29].

In 2010, patient representatives of the stakeholders’ forum of Biological and Biomolecular Resource Infrastructure (BBMRI) produced a “consultation document on patient perspectives” which was subsequently endorsed by a number of major pan-European patient organisations [30]. The document describes the relevant principles laid down in European and international instruments that cover patient participation in networked biobanking activities. Three key principles were highlighted for the governing of active participation of patients and patient organisations in biobanking activities: *Inclusion*, *Engagement* and *Communication*. Translating these key principles into practice involves:

- Inclusion of patients and patient organisations as partners in the research effort, especially in the areas of communication, advocacy and recruitment (e.g. information to potential donors, preparation of informed consent forms).
- When establishing sample, tissue and databanks, the experience, knowledge and expertise of patients, families and carers should be considered.
- Listening to patients’ voices/expectations on research needs from their experience from participating in biobanking as donors.
- Regular, general and reasonable feedback to patients regarding use, sharing and transfer of samples.

In particular, the document recommends that patient involvement should be extended to ethical aspects such as informing Research Ethics Committees (RECs) of patients’ interests, patient rights over their donation, access to samples from research groups out of the country, possible new use of existing samples and/or discontinuation of the biobank.

In 2012, the EU-FP7 PatientPartner project identified a number of patients’ needs for partnership in the context of clinical trials, including for biobanking activities. The project consulted extensively with patient organisations through a number of different engagement mechanisms and made recommendations on the role that patient organisations have in biobanking, including a need to educate patient organisations on how to start and structure a biobank [31].

#### III: Capturing patient and public perspectives and concerns

Biobanks create a number of ethical and legal issues related to research governance, privacy, informed consent [32], control and ownership [33], withdrawal of samples and consent [34], commercialisation [35], return of results and incidental findings [36]. For biobanking operators, gaining a greater understanding of the perspectives of different stakeholders, including patients and the public, can offer insight into the nature and context of these ethical challenges and can assist in public engagement.

The findings from a plethora of public consultations have shown that the public response to biobanking activities is varied, diffuse and highly context-specific [37, 38]. In general, despite largely positive attitudes towards biobank participation, the concerns and reservations expressed by patients and the public, as participants in biobanking, involve:

- the nature of consent when agreeing to participate
- the confidentiality of the tissue and of data being compromised
- access to the tissue and data after donation
- how their information might be used
- their access rights to results from research performed using their tissue and data

A common recommendation resulting from public and patient consultations is the principle of transparency—that operators of biobanks should actively communicate relevant information to patients in a clear and transparent fashion, thus striving to build a relationship of trust with participants and the wider public [30]. Transparency can be achieved through clear information being used at all times in order to foster acceptance of the ways a biobank is developed and used.

To secure the trust of patients and the public, more recently established population-based biobanks [39, 40] have conducted consultation exercises aimed at obtaining and incorporating patient and public perspectives into the design of the biobank prior to its implementation [41]. However, these exercises have not provided patients and the public with the opportunity for active involvement in running of the biobank once it was established. An exception is the Mayo Clinic Biobank [42] highlighted in the case studies in this article, which, using deliberative democracy techniques [43], has involved patients and research participants in the policy and decision-making of the biobank.

#### IV: Education and training

It is commonly accepted that in order to ensure that biobanks are developed and used to their full potential, it is essential that both researchers and other stakeholders associated with biobanking have access to the best possible training and career development opportunities at all stages of their professional life. Therefore, in order to facilitate active participation of patients and patient organisations in biobanking activities, appropriate training in partnership with healthcare professionals and researchers is and will be necessary.

A number of ongoing patient-led training initiatives in this area are helping to raise awareness of the contribution that patients can make as partners in research. The European Patients’ Academy (EUPATI), an EU-wide, public-private partnership that is led by the European Patients’ Forum and is funded by the Innovative Medicines Initiative (IMI), provides in-depth training of patient advocates as well as educational material on all aspects of medicines research and development (R&D) including key topics relevant to patient involvement in biobanking (e.g. the principles of non-clinical development, exploratory and confirmatory clinical development, informed consent processes and regulatory affairs). EUPATI’s Expert Patient Training Courses are currently training 50 patient advocates, and its Internet Library will provide this information in the form of web-based educational resources to the health-interested general public in seven languages in 2016 [12].

The Vision On Information, Confidence and Engagement (VOICE) patient training course in the UK trains patient advocates in basic cancer biology, research terminology and study design, and the critical evaluation of research proposals and scientific papers [44]. The Genetic Alliance Biobank equips patients and the public with an understanding of the opportunities and issues in becoming actively involved in biobanking through running a regular “boot camp” providing in-depth training to assist patient organisations in creating and maintaining a registry and/or biobank [45].

Equipping research professionals with the understanding to start involving patients and public as partners in biobanking and to develop practice is a distinct challenge in this area. However, there remains a potential for this training to be provided in connection with leading universities/training sites/course providers in the field (e.g. P3G, ISBER). In addition, possible topics for patient seminars, summer schools, and short/long-term fellowships in the field of biobanking research could be a valuable addition to current training programmes in biobanking [46].

## Case studies

### Biobanks established by patients

There are many examples, predominantly from the rare disease patient community, of patient stakeholders who have invested their own resources in the establishment of new biobanks and biobank infrastructures. Below are three case studies which capture the levels of involvement and the resulting value to the biobank itself.

Case study 1:

**Table:** 

Name of the biobank	Patients’ Tumor Bank of Hope (PATH Biobank)
Date established	2002
Size	Approximately 7500 patients have consented to be donors with seven sample source sites across Germany (ongoing)
Host organisation	PATH Foundation, Germany
Country or region	Germany
References	References 47 and 48 below

Breast cancer survivors established the PATH Biobank in 2002 as a non-profit organisation to collect human tumour and blood samples, together with patient data (and follow-up data) at high ethical standards and under uniform SOPs. PATH [47] aims to involve the breast cancer patients as much as possible in its work through the following strategy:

1. The current PATH board consists of three breast cancer survivors, which according to its statutes, at least two members have to be former patients. In addition to the representation (e.g. at conferences and towards scientific partners), the board guides the activities and direction of PATH Biobank. As breast cancer survivors, all board members have an inherent motivation to contribute to the cure of this disease.
2. PATH respects the proprietary right of the breast cancer patient. The patient donates their blood and tumour, and in return, PATH reserves one aliquot of both blood serum and tumour tissue, for the donor. The donor may request these samples at any time, free of charge. This way, the breast cancer patient is directly involved in the biobanking process and may benefit from the storage of her biospecimens.
3. PATH patients provide their follow-up data. In a follow-up study, PATH patients were re-contacted by telephone. More than 75 % of contacted patients provided data about their disease course and medical treatments. The extraordinarily high response rate reflects the patients’ trust and interest in PATH’s work [48].
4. PATH is transparent. PATH publishes the results of studies performed with PATH samples on the PATH website (http://www.path-biobank.org). This way, PATH patients can directly see what PATH samples have achieved. Additionally, PATH biospecimen donors receive a yearly newsletter. The newsletter covers topics like clinical studies, new therapeutics and progresses in research and development. The patients’ feedback indicates that they appreciate the newsletter. Importantly, only 31 % of all PATH patients use the internet as a source of information highlighting the important role of biobanks like PATH in patient information [48].
5. PATH consults patients free of charge concerning the handling of their tumour tissue, especially regarding their right to self-determination. In a normal clinical setting, there is no time to discuss the subsequent use of the tumour tissue. Informed patients demand to be included in these decisions. They have right to decide what happens to their tumour. For these reasons, breast cancer patients may call the PATH office and receive support free of charge. The PATH staff consists of a physician and a biologist, thus allowing for competent consultancy regarding all questions associated with the handling of the biomaterial and proprietary rights.
6. PATH encourages breast cancer patients to become active and support PATH’s work by volunteering their time. As a non-profit organisation, PATH is always looking for volunteers. This is done via the PATH newsletter, an email-newsletter and patient events.

Case study 2:

**Table:** 

Name of the biobank	Chordoma Foundation Biobank
Date established	2012
Size	110+ samples (ongoing)
Host organisation	Chordoma Foundation
Country or Region	North Carolina, USA, with four partner sites/hospitals across the USA
References	Reference 49 below

Chordoma is a rare bone cancer that can occur anywhere along the spine. The Chordoma Foundation was founded in 2007 by Josh Sommer, a then 17-year-old patient, and is a small, volunteer-run patient organisation. The Chordoma Foundation decided in 2010 to establish its own biobank [49] with the aim not only to fund and endorse the biobank but to remain in control and act as the formal “sponsor” of the research arising from the use of the tissue. The biobank is designed, supported and operated by the staff and board of the foundation, with the assistance of a Scientific Advisory Board, and third parties where appropriate (e.g. storage). The foundation operates its biobank under an institutional review board (IRB) approved research protocol.

The patient-led biobank team works closely with hospitals to coordinate chordoma tissue collection from patients, providing the first centralized source of chordoma samples and data to the research community. The foundation also developed a standardized kit to make it easy for any hospital to provide tissue samples, thus ensuring the quality of sample collection and preservation during transport.

As the condition is very rare, and the tissue so scarce, the foundation has made specific efforts to liaise closely with hospitals to collect any chordoma tissue remaining from surgical procedures that is not needed for patient care. The foundation has also set up partnership networks with hospitals that are centres of excellence for the treatment of chordoma patients to routinely collect chordoma tissue. Expanding the biobank network to Europe and exploring “virtual” biobank solutions (where specimens are administered centrally but stored locally) are currently being investigated. A major goal for the near future is to acquire more frozen samples suitable for genetic research.

Volunteers and patients are also involved in promoting the biobank to the public through the foundation’s online (social media, website) and offline (face-to-face meetings, mailings, brochures) tools and channels. Because the patient organisation is in the driver’s seat, chordoma patients are very easily convinced about the necessity of tissue donation. This has all been achieved with the support of chordoma patients and their families, acting as volunteers, and a small staff (<5). The Foundation board consists of patients or relatives of patients, in the majority. The message from the Chordoma Foundation is that patient/volunteer-led biobanking can be performed very effectively, even by very small organisations, and may be the only solution for some rare diseases.

Case study 3:

**Table:** 

Name of the biobank	I.B.AHC—Italian Biobank for Alternating Hemiplegia of Childhood
Date established	October 2004
Size	48 patients (ongoing)
Host organisation	Scientific Institute IRCCS “E. Medea”
Country or region	Bosisio Parini (LC)—Italy
References	Reference 50 below

Alternating Hemiplegia of Childhood (AHC) is a very rare, progressive and debilitating neurological disease, characterized by an early onset. The Italian Association for Alternating Hemiplegia of Childhood (AISEA) is actively involved in their Italian Biobank AHC (I.B.AHC), an open repository by which the clinical information and the biological material of AHC patients with a validated diagnosis are collected and made usable for any research project on AHC [50]. The I.B.AHC Biobank and associated clinical registry are fully integrated and have been funded and coordinated by the patient association since 2004, in association with its scientific committee.

I.B.AHC is composed of two linked repositories—a clinical registry of patient data and a biobank of DNA samples from participating patients. AISEA defined both the biobank and registry protocols together with its scientific committee and with the ethics committee of the scientific institute hosting the biobank (MEDEA, Italy). The informed consent forms for patients and the Material and Data Transfer Agreement (MDTA) for the researchers applying for access were also defined by the patient organisation in collaboration with the MEDEA Ethics Committee.

AISEA has a primary responsibility for the recruitment of AHC patients and plays a vital role in active communication with patients, particularly about the process of sample/data collection, as well as information on the communication of individual results obtained by the research projects that used their sample/data. According to conditions stated in the MDTA, AISEA enters the individual results of the research projects accessing I.B.AHC into the clinical registry, so that they can be seen and used by any other future study accessing I.B.AHC. In this way, the patients, by donating their data and samples to I.B.AHC, obtain the maximum return of their investment for the progress of research on AHC. AISEA also communicates the individual results of the studies accessing I.B.AHC to the patients and to their treating physicians.

The I.B.AHC was the largest case series included in an international collaborative research project that in July 2012 identified mutations in the ATP1A3 gene as the major cause for AHC. The I.B.AHC organisational and information model, in compliance with the model developed by ENRAH, the European Network for the Research on AHC, is now being used to create an international network of homogeneous clinical registries with linked biobanks as a supporting infrastructure for the International Consortium for the Research on AHC (IAHCRC). In this initiative, the AISEA together with other national patient associations for AHC are playing an essential role in its governance, not only for guaranteeing the sustainability of this infrastructure and the recruitment of the patients but also for the definition of rules for sharing of the data and samples inside IAHCRC.

### Patient involvement as part of biobank governance

Biobanks are increasingly incorporating patient involvement as an important part of their governance structures. Three case studies are presented below.

Case study 4:

**Table:** 

Name of the biobank	Wales Cancer Bank
Date established	2004
Size	Currently 12,000 patients (ongoing)
Host organisation	Cardiff University
Country or region	Wales, UK
References	Reference 51 below

The Wales Cancer Bank (WCB) is funded by the Welsh Government and Cancer Research Wales and is hosted by Cardiff University [51]. Patient involvement has been key to the development of the WCB since its inception and the insight given by the lay members complements and augments the expertise of the professionals involved in managing and advising the project.

A steering group was convened when the biobank was founded to ensure all relevant stakeholders were able to input into the formation and initiation of the WCB. Of the 38 steering group members, four were “lay members” recruited either through personal contact or via the local patient support groups and/or networks. Three lay members were patients, and one was a caregiver for a cancer patient. These four lay members went on to form the core of the patient and ethics committee, which drafted the ethics application, the patient information sheet and consent form and advised on the proposed patient pathways. They were instrumental in keeping the discussions grounded, ensuring the language and content of the patient documentation was suitable and giving invaluable advice regarding the timing and method of consent.

The patient and ethics committee was later reconfigured and revitalised to become the Lay Liaison and Ethics group (LLEG). The group expanded its lay membership further to include a public relations expert, a fundraising expert and additional patients. Mr. Neil Formstone, who was one of the original three patients involved in the steering group and who remained heavily involved as a committed patient advocate until his death in December 2012, initially chaired the new group. The Chair of the LLEG committee is a full member of the WCB Executive group, which is the central pillar of the governance structure responsible for the operational decisions. The Executive group reports to an overarching Advisory Board with a remit to oversee the progression and future strategy of the WCB. The lay voice is, therefore, an integral part of the Executive group and is also heard at Advisory Board meetings where the Executive group is in attendance. The Chair of the Advisory Board is also currently a lay member.

The Lay Liaison and Ethics group has expanded further with three new patient members and has concentrated on raising the profile of the biobank through their interactions with patient groups, hospital groups and other organisations, such as Rotary. The members regularly review the communication strategy and are the first port of call for WCB to explore any proposed new processes. The value WCB places on the views and advice of the members of the LLEG committee was recently demonstrated when two suggested new procedures were first presented to the group prior to higher level discussion at the Advisory Board. Electronic consenting and volunteer consenting were both (individually) submitted to the group and only with their support have both schemes been progressed further for more detailed investigation and potential implementation.

A sub-committee of members of the Lay Liaison and Ethics group has started to review the lay summaries on the applications that the WCB receives for samples to ensure that they are understandable to a general audience. Researchers will not receive samples until the reviewing LLEG member is satisfied that the content and structure of the lay summary describes the proposed research in terminology suitable for a lay audience. These summaries are uploaded onto the WCB website and feature in the annual report to publicise the projects supported by the collection.

Case study 5:

**Table:** 

Name of the biobank	The Mayo Clinic Biobank
Date established	2009
Size	The target goal is to obtain 50,000 biospecimens (ongoing)
Host organisation	Mayo Clinic, Rochester
Country or region	Minnesota, USA
References	Reference 52 below

Following a deliberate community engagement event in September 2007, patient and public participants created recommendations for the design of the Mayo Clinic Biobank [52]. The hopes and values expressed guided Mayo Clinic’s development of the biobank, particularly its procedures and practices to protect the individuals donating to the biobank. One recommendation voiced by the participants was the need for ongoing community guidance and involvement in biobank governance. Therefore, Mayo Clinic established a Community Advisory Board (CAB) to ensure that the voice of the community continues to be heard. The Mayo Clinic Biobank CAB is composed of approximately 20 members chosen to reflect the diversity of community interests and backgrounds, several of whom participated in the Biobank’s deliberative community engagement event. The CAB provides community input to the biobank leadership about current and future plans for maintenance and growth. Whilst the recommendations provided by the CAB members to the leadership are not binding, they are viewed with considerable respect and are often incorporated into policies, actions and decisions that leadership make. The CAB is co-chaired by a community member (elected by CAB members) and a member of the Mayo Clinic Biomedical Ethics Program. The co-chairs work with Biobank staff to set meeting agendas and facilitate CAB meetings. Both co-chairs are also active voting members of the Biospecimen Trust Oversight Group and Biobank Access Committee. A community vice chair (elected by CAB members) is also part of CAB leadership and helps with co-chair responsibilities. In 2008, CAB members met with Mayo Clinic Biobank staff members to assist in creating the Biobank’s informed consent procedures and review recruitment materials and methods. Many of the improvements and suggestions generated by the CAB were adopted by the Mayo Clinic Biobank before it began operations in April 2009. Since then, the Biospecimen Trust Oversight Group has asked the CAB to review and recommend policies and procedures on issues arising in Biobank research, such as return of research results, data sharing with other researchers, procedures used to recruit Biobank donors and many other topics. The CAB meets every other month to learn about, discuss and develop recommendations to help guide the Biobank leadership.

Case study 6:

**Table:** 

Name of the biobank	Nottingham Health Science Biobank
Date established	January 2011
Size	The aim is to consent every patient at the time of first presentation at our hospital and those referred for surgery and treatment after diagnosis (ongoing)
Host organisation	Nottingham University NHS Hospitals Trust
Country or region	Nottingham, UK
References	Reference 53 below

The Nottingham Health Science Biobank (NHSB) has pioneered an innovative model of patient-led consent which is being piloted and developed with the help of the Biobank PPI Advisory Group, comprising volunteer patients, carers and the public [53]. In this model, patients themselves lead the consent process for biobanking. Many of the patient volunteers have experience of the diseases the NHSB is researching. A key innovation of this model is helping to build lasting relationships with an important stakeholder that biobanks often neglect—the patient donors of the tissue samples.

Nottingham University Hospitals have 1500 PPI members on their database. To engage the volunteers, an expression of interest was sent to 200 PPI members outlining what the NHSB did, why it was set up and the activities that would be requested for PPI members to become involved in. These activities included the design development and delivery of innovative, effective and user-friendly consenting processes to encourage improved donation activity, delivery of easy-to-understand information for patients and the public, and provision of greater opportunities to share biobank-related news with patients and the public via various media sources on a local, regional and national level. As part of the process, a comprehensive job description was created including the nature of the role for a person consenting, time commitment, support and reporting, confidentiality, probationary period and length of membership. A comprehensive “person specification” also outlined the essential and desirable skills for experience, special attributes and communication skills. From this process, 25 people who were interested in the NHSBs’ activities were identified and invited to a face-to-face meeting. After further consultation with the PPI advocates, nine wished to take part in the consent process for the NHSB. PPI members who wished to take part:

- received full mandatory training and induction by the Hospital Trust
- were given an Honorary Trust contract
- had to sign a confidentiality agreement
- were required to have a Criminal Records Bureau (CRB) check and engage with the Independent Safeguarding Authority (ISA) to carry out the Disclosure and Barring Service (DBS)
- were provided with Hospital badges
- were given free car parking permits
- were reimbursed for travel expenses

In addition, the NHSB also produced a comprehensive consent training package which included:

- a presentation followed by role plays
- hand-holding in the clinic for a minimum of six clinics. This aspect of the training was tailored to the individual and no time limits were set
- shadowing and review
- direct observation and final competency sign offs

Each advocate was also provided with one-to-one training and taken through the full life cycle of biobanking. As part of their ongoing professional development, the PPI advocates are also offered annual appraisals. NHSB have now recruited five PPI Advocates, all of whom have had breast cancer or are the partners of people with a history of breast cancer, to lead the process of consent in this specialist area. Since August 2012, the PPI advocates have been responsible for consenting in five out-patient breast clinics. The new role has had excellent feedback from both patient and the advocates and has led to increases in the rates of consent.

The use of PPI advocates to take consent is cost-effective and embeds the patient perspective at the core of the biobanking process. According to NHSB, this is the first description of a novel and broadly applicable approach to consent for biobanking. In addition, the current PPI advocates have expressed an enthusiasm to be involved in training of future biobank volunteers. The NHSB continues to promote PPI and patient and public engagement (PPE) through regular group meetings, open days and speciality targeted workshops.

## Conclusions

Reflecting a “cultural revolution” in health research over the last two decades, we believe that PPI in biobanking practices and research is here to stay. PPI represents an ideological shift within which patients can take a more central, driving role in research that affects their health and healthcare.

This article provides examples of the variety of ways that active patient involvement has been conducted to date. It also provides evidence to support the importance of early and sustained engagement and involvement of patient representatives in biobanks. As more and more biobanks are created in the future, so too will the number of patients and patient organisations that get involved in biobanking, as donors, as active participants and collaborators in research.

To facilitate this increased role as collaborators in biobanking, appropriate training is necessary and more information should be disseminated as to how patients can concretely contribute to biobank-based research.

In-depth training and educational resources are now available, for example, through the EUPATI’s Expert Patient Training Courses, the EUPATI Internet Library—becoming available in 2016 in seven languages, as well as the Cochrane’s “Consumers for Evidence-Based Healthcare” initiative [54]. In addition, as biobanks in general are evolving from the stand-alone, proprietary model to more networked, harmonized models, there is now a significant opportunity to assess the extent of patient and public involvement across all biobanks and to include the PPI perspective in international initiatives of biobanking harmonisation like BBMRI and ISBER.

**Fig. 1:**
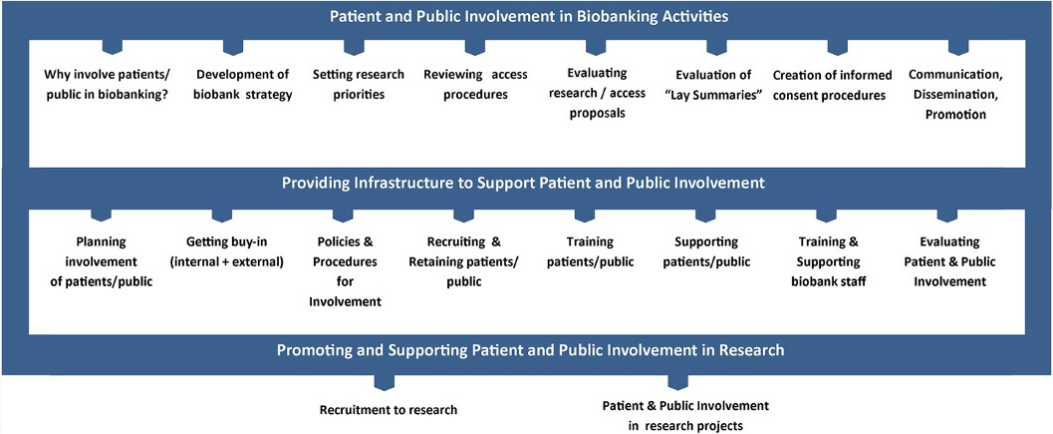
Potential roles, supportive infrastructures and likely outcomes of PPI in biobanking activities. Adapted with permission from TwoCan Associates [55]

Organising and facilitating PPI activity in biobanking takes considerable time and effort for all stakeholders involved. Therefore, for any biobank operator considering involving patients in their biobanking activities, consideration of best practices, current guidance, ethical issues and evaluation of involvement will be important. To act as reference guide in this regard, the examples of involvement outlined in this article are summarized and illustrated in Fig. 1, with potential roles, supportive infrastructures and likely outcomes for PPI in biobank-based research highlighted.
